# Safety and immunogenicity of a Herpes Zoster subunit vaccine in Japanese population aged ≥50 years when administered subcutaneously vs. intramuscularly

**DOI:** 10.1080/21645515.2016.1232787

**Published:** 2016-12-09

**Authors:** Peter Vink, Masanari Shiramoto, Masayuki Ogawa, Masahiro Eda, Martine Douha, Thomas Heineman, Himal Lal

**Affiliations:** a GSK Vaccines, Rockville, MD, USA; b SOUSEKAI PS Clinic, Tenyamachi, Hakata-ku, Fukuoka, Japan; c Japan Vaccine Company Ltd., Chiyoda-ku, Tokyo, Japan; d GSK Vaccines, Wavre, Belgium

**Keywords:** adjuvant, adjuvanted vaccine, herpes zoster, HZ/su vaccine, immunogenicity, intramuscular, subcutaneous, reactogenicity

## Abstract

The impact of alternate routes of vaccine administration, subcutaneous (SC) or intramuscular (IM), on the safety and immunogenicity of herpes zoster subunit candidate vaccine (HZ/su) was assessed in Japanese adults aged ≥ 50 y. During this phase III open-label study, 60 subjects were randomized (1:1) to receive HZ/su through SC or IM routes in a 0, 2 month schedule. Vaccine response rates (VRRs) and geometric mean concentrations (GMCs) of varicella zoster virus glycoprotein E (gE)-specific antibodies were determined by ELISA. Solicited and unsolicited symptoms were recorded for 7 and 30 d after each vaccination and graded 1–3 in severity. Serious adverse events (SAEs) were recorded throughout the study. At one month post-dose 2, VRRs were 100% (95% Confidence Interval (CI): 88.1–100) in both groups; anti-gE antibody GMCs were 44126.1 mIU/ml (95% CI: 36326.1–53601.0) and 45521.5 mIU/ml (95% CI; 37549.5–55185.9) in the SC and IM groups, respectively. Injection site reactions (pain, swelling and redness) were common, and observed more frequently following SC administration. Grade 3 redness and swelling were more frequently observed after SC administration. Fatigue and headache were the most frequently reported general symptoms for both routes of administration. Ten and 7 unsolicited AEs were reported in the SC and IM group, respectively. Two unsolicited AEs (1 in SC; 1 in IM) were considered related to vaccination by the investigator. Three non-fatal SAEs considered unrelated to vaccination were reported during the study. Administration of the HZ/su vaccine candidate resulted in a substantial immune response that was comparable between SC and IM subjects, but local reactogenicity may be greater for SC.

## Introduction

Herpes Zoster (HZ) is caused by the reactivation of latent varicella zoster virus (VZV) in the dorsal root or cranial nerve ganglia.^1^ Primary VZV infection causes varicella (chickenpox) and usually occurs in childhood, after which the virus lays dormant in the affected ganglia, often for decades, until reactivation.^2^ HZ is typically characterized by a vesicular rash in a unilateral dermatomal distribution that is usually accompanied by radicular pain.^1^ The incidence of HZ increases with age,^2-4^ at least partly due to age-related decline in VZV-specific immunity.^5^ HZ risk begins to increase particularly in adults ≥ 50 y of age (YOA).^6^ In Japan, HZ incidence has been reported to be 4.15 per 1,000 person-years,^7^ comparable to the incidence rates reported in the US and Europe.^8-11^

The herpes zoster recombinant subunit candidate vaccine (HZ/su), consisting of VZV glycoprotein E (gE)^12^ and the proprietary AS01_B_ adjuvant system,^13^ when administered intramuscularly (IM) has shown an age-independent vaccine efficacy of 97% in the prevention of HZ, with a clinically acceptable safety profile.^14^ In Japanese subjects, IM administration of 2 doses of HZ/su resulted in a substantial immune response,^15^ comparable to other study populations.^16-18^

In some countries, notably Japan, subcutaneous (SC) vaccine administration is preferred over IM vaccination.^19^ As previous investigations with other vaccines have suggested that both vaccine immunogenicity and reactogenicity may be impacted by the route of administration,^20-21^ this study was conducted to evaluate the safety and immunogenicity of the HZ/su candidate vaccine in Japanese adults 50 YOA or older when HZ/su was administered SC compared to IM.

## Results

### Demographics

**Figure 1.**
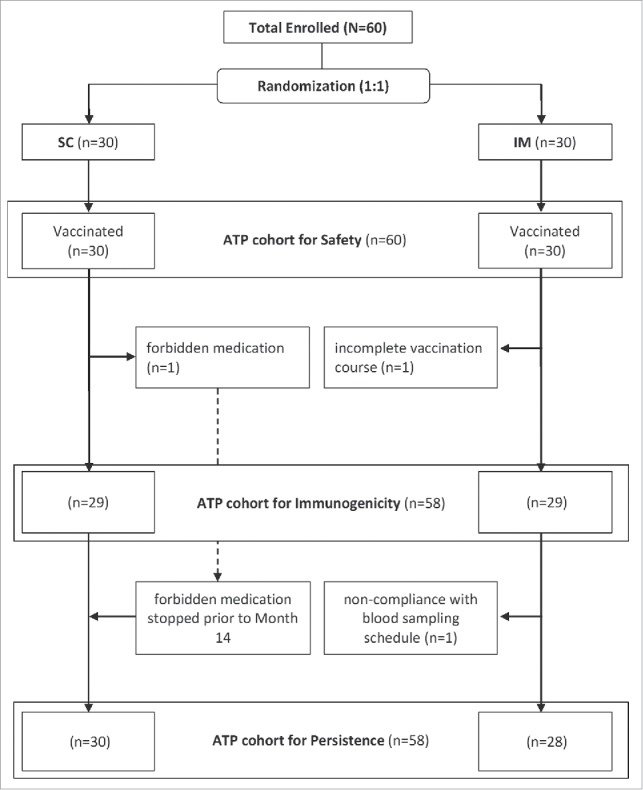
Disposition of study participants. SC: Subcutaneous IM: Intramuscular.

A total of 60 subjects were enrolled in the study (30 subjects each in the SC and IM groups; Fig. 1). In both groups, 24 (40%) subjects were 50–59 YOA, 24 (40%) were 60–69 YOA and 12 (20%) were at least 70 YOA. The mean age of subjects was 61.9 y (standard deviation: 7.7), and 50% of subjects were female.

### Immunogenicity

**Figure 2.**
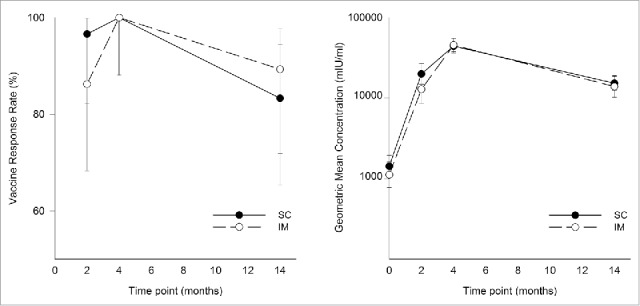
Vaccine response rate and geometric mean concentrations of anti-gE antibody (ATP cohort for immunogenicity). Samples were collected at the indicated time points (for both subcutaneous (SC) versus intramuscular (IM) groups) and anti-gE antibody concentrations were determined by ELISA. Data are vaccine response rates (VRRs) and geometric mean concentrations (GMCs) and error bars indicate 95% confidence interval.

All subjects were seropositive for anti-gE antibodies at study entry. At one month post-dose 2, Vaccine Response Rates (VRRs) were 100% [95% Confidence Interval (CI): 88.1 – 100]) for both SC and IM groups. At 12 months post-dose 2, the percentage of subjects above the VRR cut-off declined, but was still comparable between groups (SC: 83.3% [95% CI: 65.3 – 94.4]; IM: 89.3% [95% CI: 71.8 – 97.7]; Fig. 2).

The mean geometric increase (MGI) was 31-fold (SC group) and 40.8-fold (IM group) at one month post-dose 2, and 10.7-fold (SC group) and 11.6-fold (IM group) at 12 months post-dose 2 (Fig. 2). At one month post-dose 2, anti-gE antibody Geometric Mean Concentrations (GMCs) were 44126.1 mIU/ml (95% CI: 36326.1–53601.0) and 45521.5 mIU/ml (95% CI; 37549.5–55185.9) in the SC and IM groups, respectively. At 12 months post-dose 2, anti-gE antibody GMCs were 15250.9 mIU/ml (95% CI: 12464.0–18660.9) and 13870.2 (95% CI: 10184.2–18890.3), in the SC and IM groups, respectively.

### Safety and reactogenicity

**Figure 3.**
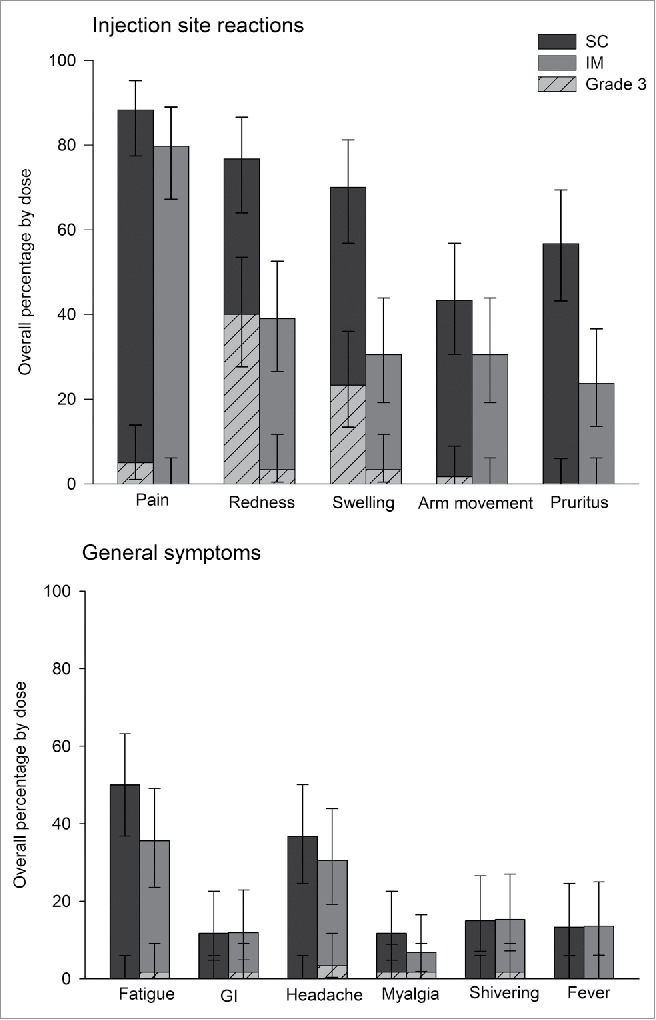
Overall occurrence of solicited injection site reactions and general symptoms during the 7-day vaccination follow-up period (Total vaccinated cohort).

During the 7-days post-vaccination, at least one solicited or unsolicited symptom was reported after 100% of doses in the SC group and 91.5% of doses in the IM group. Solicited injection site reactions were common, and were reported after 98.3% of doses in the SC group and after 84.7% of doses in the IM group. Pain was the most frequently reported injection site reaction after both SC (88.3% of doses) and IM (79.7% of doses) administration. Redness and swelling were more frequent after SC (76.7% and 70% of doses) compared with IM (39% and 30.5% of doses) administration (Fig. 3).

Solicited systemic symptoms were reported after 60% of doses in the SC group and 50.8% of doses in the IM group. Fatigue was the most frequently reported general symptom for both SC and IM administration, observed after 50% of SC doses and 35.6% of IM doses. Grade 3 solicited systemic symptoms were uncommon, with Grade 3 myalgia reported most frequently in the SC group (after 1.7% of doses) and Grade 3 headache reported most frequently in the IM group (after 3.4% of doses) compared to SC group (0%) (Fig. 3).

During the 30 d following vaccination, 10 unsolicited adverse events (AEs) were reported by 9 subjects in the SC group, and 7 unsolicited AEs were reported by 6 subjects in the IM group. No specific AE was reported by more than one subject. Seven subjects in the SC group and 3 subjects in the IM group experienced at least one unsolicited symptom necessitating a medically attended visit. Nasopharyngitis [1 subject in SC group] and pollakiuria [1 subject in IM group] were the only 2 unsolicited AEs that were considered related to vaccination by the investigator.

Two subjects in the SC group and one subject in the IM group experienced a non-fatal serious adverse events (SAEs) during the study period (SC: chronic obstructive pulmonary disease exacerbation 6 d after dose 2, finger deformity after 271 d post-dose 2; IM: spinal compression fracture after 360 d post-dose 2). These SAEs were not considered to be causally related to the study vaccine by the investigator. No HZ cases or potential immune mediated diseases were reported during the study period.

## Discussion

This study assessed the impact of alternative routes of vaccine administration on the immunogenicity and safety of the candidate HZ/su, in Japanese adults aged ≥50 years. Immune responses were comparable between SC and IM routes of administration. However, injection site reactions of any grade, and Grade 3 redness and swelling in particular, were observed more frequently after SC administration.

The HZ/su candidate vaccine has consistently been shown to elicit robust immune responses in humans including in adults aged 50 y and older.^16-18^ The current results are consistent with previous findings and further expand those findings by suggesting that immunogenicity of the vaccine is independent of the route of administration.

Although it has been suggested that IM administration contributes to a stronger immune response,^22,23^ our study would be in line with studies of other vaccines that report immune responses to be unaffected by the route of administration.^24-27^

Injection site reactions to HZ/su were observed more frequently after SC administration. This effect was limited to local symptoms; in particular, redness and swelling, and we found no evidence for a difference in systemic symptoms between routes of administration. This is also consistent with studies of other vaccines.^20-24^

Therefore, although the small sample size limits the extent to which these results can be extrapolated to the general population, in combination with the body of research already available, this study suggests that SC administration of the HZ/su candidate vaccine is unlikely to affect immunogenicity, but local reactogenicity may be greater.

## Methods

### Study design and subjects

This phase III, open-label, randomized (1:1) single-center trial (ClinicalTrials.gov identifier NCT01777321) was carried out in Japan, between 17 June 2013 and 11 November 2014. Healthy subjects of Japanese ethnic origin, aged 50 y or older, were enrolled. Subjects received 2 doses, with a 2 months interval, of the HZ/su vaccine in the deltoid region either by SC or IM route. As this was the first time the HZ/su candidate vaccine was administered SC in humans, a GSK Safety review committee (SRC) reviewed all safety and reactogenicity data accrued during a 7-day period following the first dose. The SRC review included consideration of prespecified criteria for acceptable frequency and severity of AEs, and the second dose was administered only after approval of the SRC.

Potential subjects were excluded from participation if they received any investigational or non-registered drug/vaccine within 30 days, or any immunosuppressants or immune-modifying drugs within 6 months before study start, were allergic to any vaccine component, had a history of HZ, or were previously vaccinated against HZ or varicella. Other reasons for subject exclusion were any underlying illness, pregnancy, or planning to get pregnant. Enrollment of potential subjects with a temperature of ≥37.5 was deferred until resolution of the fever.

The guidelines of the Declaration of Helsinki, Good Clinical Practice and other applicable regulatory requirements were followed while conducting the study. The study was approved by the Kyushu Clinical Pharmacology Research Clinic institutional review board, and all subjects provided written informed consents before being included in the trial.

### Study vaccine

Each dose (0.5ml) of HZ/su contained 50 μg of recombinant VZV gE combined with the AS01_B_ Adjuvant System (liposome, 50μg 3-O-desacyl-4′-monophosphoryl lipid A, 50μg of *Quillaja saponaria* Molina, fraction 21 (QS21, Licensed by GSK from Antigenics Inc., a wholly owned subsidiary of Agenus Inc., a Delaware, USA corporation). The reconstituted vaccine was administered within 6 hours of reconstitution.

### Immunogenicity assessment

Blood samples were collected before vaccination, 2 months post-dose 1, and one and 12 months post-dose 2. Antibodies against gE were measured by ELISA. The assay cut-off was 18 mIU/ml for all time-points, except for the persistence time-point at 12 months post-dose 2, for which the assay cut-off was 97 mIU/ml.

### Safety and reactogenicity assessment

Solicited injection site reactions (pain, swelling, redness, pruritus at the injection-site, and impaired movement/range of motion of the vaccinated arm) and solicited systemic symptoms (fatigue, fever, gastrointestinal symptoms [nausea, vomiting, diarrhea and abdominal pain], headache, myalgia, and shivering) were recorded for 7 d after each vaccination. All unsolicited AEs were recorded for 30 d after each vaccination. All SAEs were recorded throughout the study period. Study withdrawals and medical conditions occurring during the course of the trial were also recorded. The severity of symptoms was graded on a scale of 0–3. Grade 3 AEs were defined as “preventing normal daily activity,” with the exception of redness and swelling, for which Grade 3 was defined as a reaction with a diameter >100 mm, and fever, for which Grade 3 was defined as an axillary temperature >39°C.

### Statistical analysis

The first and second co-primary objectives were the assessment of VRRs and GMCs of anti-gE antibodies one month after administration of the second vaccine dose. A vaccine response was defined as a 4-fold increase in post-vaccination antibody concentrations as compared to pre-vaccination antibody concentrations. GMCs were calculated by taking the anti-log of the mean of the log concentrations. A MGI was calculated as the geometric mean of the within-subject ratio of the post-vaccination antibody concentration to the pre-vaccination concentration.

The third co-primary objective was the assessment of the safety and reactogenicity of the vaccine in all subjects. The percentage of subjects/doses reporting solicited injection site reactions, solicited systemic symptoms, and unsolicited symptoms were calculated with exact 95% CI.

For each endpoint, no formal comparison between groups was made, and CI were used to suggest comparability between groups. All statistical analyses were performed using the Statistical Analysis Sytems software version 9.2 (Cary, NC, USA). Immunogenicity analyses were performed on the according-to-protocol cohort for immunogenicity, which included subjects who complied with all protocol-defined study procedures and for whom immunogenicity data were available at one month post-dose 2. In order to assess the persistence of anti-gE antibodies, VRRs and GMCs with 95% CI were calculated in the ATP cohort for persistence, consisting of subjects who complied with all protocol defined study procedures and for whom the immunogenicity data were available at 12 months post-dose 2. Safety and reactogenicity were analyzed on the total vaccinated cohort which included all subjects who received at least one study vaccine dose.

## Abbreviations

AEs: Adverse events
CI: Confidence interval
gE: Varicella zoster virus glycoprotein E
GMCs: Geometric mean concentrations
HZ: Herpes zoster
HZ/su: Herpes zoster subunit vaccine
IM: Intramuscular
MGI: Mean geometric increase
SAEs: Serious adverse events
SC: Subcutaneous
SRC: Safety review committee
VRRs: Vaccine response rates
VZV: Varicella zoster virus
YOA: Years of age

## Notes

**Clinical Trial Registration**: www.clinicaltrials.gov, NCT01777321
